# Liver function tests in primary care provide a key opportunity to diagnose and engage patients with hepatitis C

**DOI:** 10.1017/S0950268822000978

**Published:** 2022-06-27

**Authors:** A. McLeod, S. J. Hutchinson, A. Weir, S. Barclay, J. Schofield, C. Gillespie Frew, D. J. Goldberg, M. Heydtmann, E. Wilson-Davies

**Affiliations:** 1 Clinical and Protecting Health Division, Public Health Scotland, Glasgow, UK; 2 School of Health and Life Sciences, Glasgow Caledonian University, Glasgow, UK; 3 Department of Gastroenterology, Glasgow Royal Infirmary, Glasgow, UK; 4 Department of Sociology, Social Police and Criminology, University of Stirling, Stirling, UK; 5 West of Scotland Specialist Virology Centre, Glasgow Royal Infirmary, Glasgow, UK; 6 Department of Gastroenterology, Dumfries & Galloway Royal Infirmary, Cargenbridge, UK; 7 Southampton Specialist Virology Centre, University Hospital Southampton NHS Trust, Southampton, UK

**Keywords:** Epidemiology, hepatitis C, public health, surveillance, virology (human) and epidemiology

## Abstract

Since the advent of direct-acting antiviral therapy, the elimination of hepatitis c virus (HCV) as a public health concern is now possible. However, identification of those who remain undiagnosed, and re-engagement of those who are diagnosed but remain untreated, will be essential to achieve this. We examined the extent of HCV infection among individuals undergoing liver function tests (LFT) in primary care. Residual biochemistry samples for 6007 patients, who had venous blood collected in primary care for LFT between July 2016 and January 2017, were tested for HCV antibody. Through data linkage to national and sentinel HCV surveillance databases, we also examined the extent of diagnosed infection, attendance at specialist service and HCV treatment for those found to be HCV positive. Overall HCV antibody prevalence was 4.0% and highest for males (5.0%), those aged 37–50 years (6.2%), and with an ALT result of 70 or greater (7.1%). Of those testing positive, 68.9% had been diagnosed with HCV in the past, 84.9% before the study period. Most (92.5%) of those diagnosed with chronic infection had attended specialist liver services and while 67.7% had ever been treated only 38% had successfully cleared infection. More than half of HCV-positive people required assessment, and potentially treatment, for their HCV infection but were not engaged with services during the study period. LFT in primary care are a key opportunity to diagnose, re-diagnose and re-engage patients with HCV infection and highlight the importance of GPs in efforts to eliminate HCV as a public health concern.

## Introduction

With the introduction of direct-acting antiviral (DAA) therapy, with high efficacy, minimal side-effects and all-oral regimens, elimination of hepatitis C virus (HCV) is now possible [1, 2]. Scotland has recently committed to an accelerated programme for the elimination of HCV as a major public health concern by 2024 [3]. To achieve this, the government target for the number of individuals initiated onto treatment will increase to 3000 per year. Identification of those who remain undiagnosed, and re-engagement of those who were diagnosed in the past and remain untreated, is essential to meet these targets and the elimination goal.

Primary care is a key setting for HCV case-finding and re-engagement particularly of an older cohort born in the 1950s to 1970s [4, 5]. Targeted screening studies within these GP practices in areas of high drug use prevalence and deprivation have demonstrated successful increases in both testing and diagnoses [4–6]. HCV disproportionately affects those living in areas of deprivation with half of all individuals diagnosed residing in the most deprived Scottish Index of Multiple Deprivation (SIMD) populations-weighted quintile [7]. A survey of GPs in Scotland in 2007 and 2013 found that while most would not actively seek out risk factors for HCV, the majority would offer a test to patients with abnormal liver function test (LFT) results [8]. Testing of patients with abnormal LFTs, specifically raised alanine aminotransferase (ALT), has identified higher HCV prevalence than can be found in the general population in studies outside of Scotland [9–11].

The aim of this study was to determine the extent of HCV infection among individuals undergoing LFTs – both with and without an abnormal result – in primary care, considering in particular those of older age residing in areas of high deprivation. A novel design was used to estimate both the prevalence of infection and uptake of HCV testing and treatment for a large cohort of patients undergoing LFTs in primary care in the new DAA era, using a combination of unlinked anonymous HCV testing of residual sera and record-linkage to centrally held laboratory and clinical data. Thus this approach yielded insight to not just the extent of infection requiring treatment but also the effectiveness of current testing policy – specifically relating to the offer of a test to those with an otherwise unexplained elevated ALT – to identify and engage those infected. Our findings will aid the development of HCV policy and practice to improve the diagnosis and treatment of HCV infected individuals within primary care in the era of DAAs.

## Methods

### Study population

The study population consisted of 6007 patients over the age of 18 years who had venous blood collected in primary care for the purpose of LFTs in NHS Greater Glasgow & Clyde (GGC) board between July 2016 and January 2017 and for whom a residual biochemistry sample was available to retrospectively and anonymously test for HCV antibody. NHS GGC is a geographic subdivision of the health service, with a population of 1.14 million residents and accounts for a third of all HCV diagnoses in Scotland [4, 5, 7, 12]. Samples were identified from a download of biochemistry data, including sex, age (which was categorised as 18–36, 37–50, 51–65, 65+ years), the ALT result (<30, 30–49, 50–69, 70+) and GP practice where the tests were requested. AST to Platelet Ratio Index (APRI), used to predict cirrhosis without the need for biopsy or fibroscan, was calculated for patients who had an AST and platelet count result available and categorised as <1 (likely non-cirrhotic) and 1+ (likely cirrhotic) [13]. SIMD quintiles for the GP practice locations were recoded as ‘most deprived quintile’ and ‘quintiles 2–5’.

As a large proportion of LFTs from primary care is conducted on those aged 65+ years and in practices located in more affluent areas (see Online Appendix 1), samples were selected for anonymous HCV testing ensuring that the majority (~75%) related to practices in the most deprived SIMD quintile and patients aged 35 to 64 years at the time of testing. Further, as the minority of LFTs result in an elevated ALT (Online Appendix 1), samples were selected to ensure an equal split with ALT <50 U/l (regarded a normal LFT result) and >50 U/l (regarded an abnormal LFT result). Duplicate samples from the same patient were excluded. Data presented in online Appendix 1 were generated from an anonymised download of all adults (aged 18 years and older) undergoing LFTs between July and December 2016, to act as a denominator for comparison against characteristics of the study cohort.

### Unlinked anonymous HCV testing

Following identification of samples for inclusion in the study, the sample was transferred to the West of Scotland Specialist Virology Centre, assigned a random number to identify the sample and stored in a −80 °C freezer within the containment level 2 laboratory. HCV antibody testing was undertaken using the Abbot Architect platform (Abbott Laboratories, Diagnostics Division, Abbott Park, IL) and results entered onto the study dataset.

### Record-linkage to HCV laboratory and clinical databases

A separate dataset of the study population – including above characteristics (sex, age group, SIMD, ALT and APRI) plus patient initials, date of birth and sex – were provided to Public Health Scotland (PHS) for the purpose of record-linkage to HCV surveillance data. HPS maintains databases of all HCV diagnoses in Scotland since 1991, all HCV tests conducted in four NHS boards (including NHS Greater Glasgow and Clyde) since 2000, and attendance and treatment at specialist viral hepatitis clinics in Scotland since the early 1990s [14–16]. Data were matched deterministically based on the limited identifiers available. A random number was assigned to each record and patient identifiable data were then removed from the data set for analysis.

From linkage to the HCV diagnosis database, HCV diagnosis status for the study population was categorised as ‘Ever HCV Diagnosed’ then sub-divided as ‘Diagnosed 1991–2015’ (i.e. before the study period) and ‘Diagnosed 2016–17’ (i.e. during or following the study period). Those who had ever had a positive HCV PCR result were flagged as ‘diagnosed with chronic HCV’. From the HCV clinical database, information on the cascade of care was available. ‘Ever attended specialist services’ was defined as having ever attended a specialist liver clinic appointment. ‘Ever treated for HCV’ was defined as ever completing a course of HCV antiviral therapy. ‘Achieved SVR’ was defined as having completed treatment and receiving a negative PCR test result 6 months following. From the HCV test database, ‘Tested for HCV during the study period (2016–17)’ was defined as receiving any HCV test during 2016 or 2017.

Ethics approval for the testing of residual samples was granted by East Midlands – Leicester Central Research Ethics Committee and Caldicott Guardian approval was obtained for linkage to HPS data.

### Data analysis

Seroprevalence of HCV was evaluated across key characteristics (sex, age group, deprivation of locality of the GP practice, ALT level, APRI score). For HCV positive individuals, proportion HCV diagnosed was calculated and stratified by first diagnosed 1991–2015 and first diagnosed 2016–17, proportion attending services, treated for HCV and achieving SVR were also calculated. For patients with an ALT result of 30 or greater, the proportion being tested for HCV during 2016–17 was calculated. Characteristics of the study population were compared against a denominator of individuals undergoing LFTs during July to December 2016.

Data were managed and analysed using the Statistical Package for Social Sciences (SPSS) Version 24.0.

## Results

### Prevalence of HCV antibody

The study population consisted of 6007 patients who had LFT in NHS GGC during July 2016 to January 2017. In line with the study design, the majority had attended a practice (4668, 77.7%) in the most deprived quintile and were aged 37 to 64 years (4595, 76.5%) and half the cohort had an ALT level of above 50; this compares to 41.5%, 46.7% and 7.6% of all adults tested through primary care in NHS GGC during July to December 2016, respectively (Online Appendix 1). The prevalence of HCV IgG antibodies (providing evidence of past infection) in the study population was 4.0% (241/6007) (Table 1). Anti-HCV prevalence was highest for males (5.0%), those aged 37–50 years (6.2%), those with an ALT of 70 or greater (7.1%) and those with an APRI score of one or greater (10.8%).

**Table 1.** Prevalence of anti-HCV and extent of diagnosed infection, as well as uptake of specialist care and treatment for their HCV, among the study population undertaking a liver function test in primary care, during 2016–2017^a^

	Study population (2016–2017)		With anti-HCV (2016–2017)		Ever Diagnosed with anti-HCV (during 1991–2017)		First diagnosed with anti-HCV (1991–2015)		First diagnosed with anti-HCV (2016–2017)		Diagnosed with Chronic HCV (1991–2017)		Ever attended specialist HCV services		Ever treated for HCV		Achieved SVR	
	N	%	N1	% of N	N2	% of N1	N3	% of N2	N4	% of N2	N5	% of N2	N6	% of N5	N7	% of N6	N8	% of N7
Total	6007	100.0%	241	4.0%	166	68.9%	141	84.9%	25	15.1%	144	86.7%	133	92.4%	90	67.7%	55	61.1%
Sex																		
Female	2832	47.1%	82	2.9%	58	70.7%	46	79.3%	12	20.7%	51	87.9%	45	88.2%	34	75.6%	20	58.8%
Male	3175	52.9%	159	5.0%	108	67.9%	95	88.0%	13	12.0%	93	86.1%	88	94.6%	56	63.6%	35	62.5%
Age Group (in years)																		
18–36	943	15.7%	34	3.6%	22	64.7%	20	90.9%	2	9.1%	18	81.8%	17	94.4%	8	47.1%	6	75.0%
37–50	2116	35.2%	131	6.2%	109	83.2%	93	85.3%	16	14.7%	94	86.2%	85	90.4%	57	67.1%	36	63.2%
51–64	2479	41.3%	68	2.7%	32	47.1%	26	81.3%	6	18.8%	29	90.6%	28	96.6%	22	78.6%	12	54.5%
65+	468	7.8%	8	1.7%	3	37.5%	2	66.7%	1	33.3%	3	100.0%	3	100.0%	3	100.0%	1	33.3%
Deprivation status for the locality of the GP practice^b^																		
Quintile 1 (most deprived)	4668	77.7%	189	4.0%	127	67.2%	108	85.0%	19	15.0%	110	86.6%	100	90.9%	71	71.0%	40	56.3%
Quintiles 2–5	1321	22.0%	42	3.2%	29	69.0%	23	79.3%	6	20.7%	24	82.8%	23	95.8%	13	56.5%	10	76.9%
ALT Level																		
<30	2310	38.5%	53	2.3%	33	62.3%	32	97.0%	1	3.0%	25	75.8%	23	92.0%	16	69.6%	14	87.5%
30–49	700	11.7%	25	3.6%	15	60.0%	14	93.3%	1	6.7%	14	93.3%	12	85.7%	9	75.0%	5	55.6%
50–69	1756	29.2%	75	4.3%	55	73.3%	48	87.3%	7	12.7%	49	89.1%	44	89.8%	27	61.4%	15	55.6%
70+	1241	20.7%	88	7.1%	63	71.6%	47	74.6%	16	25.4%	56	88.9%	54	96.4%	38	70.4%	21	55.3%
APRI Score																		
<1	3012	50.1%	99	3.3%	71	71.7%	62	87.3%	9	12.7%	60	84.5%	54	90.0%	33	61.1%	23	69.7%
1+	380	6.3%	41	10.8%	34	82.9%	27	79.4%	7	20.6%	31	91.2%	29	93.5%	18	62.1%	9	50.0%

a Data on prevalence of anti-HCV in the study population determined through unlinked anonymous testing, while all other data determined through record-linkage of the study population to national HCV diagnosis and clinical databases.

b Deprivation measured in quintiles according to the Scottish Index of Multiple Deprivation.

**Fig. 1.**
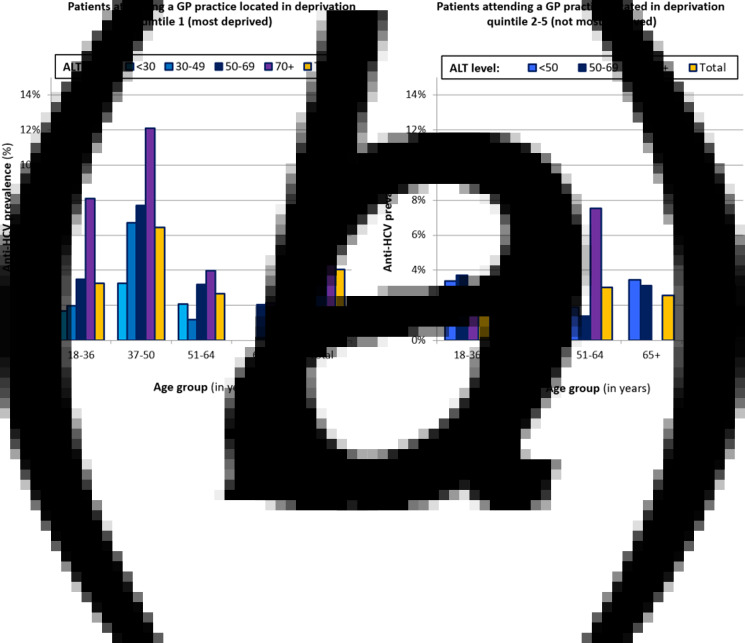
Prevalence of anti-HCV among the study population undertaking a liver function test in primary care during 2016–2017, according to age and ALT level of the patient and stratified by deprivation status of the GP practice.

For those attending GP practices in the most deprived SIMD quintile, HCV prevalence was highest for those aged 37–50 years (ranging from 3.2% for ALT <30, to 12.1% for ALT 70+) and exceeded 2% with ALT 50+ across all age groups (Fig. 1). Similarly, the highest observed prevalence in the other SIMD areas were among those with ALT 70+, 7.5% in those aged 51–64 and 6.2% in those aged 37–50 (Fig. 1).

### Extent of diagnosed HCV infection, attendance at specialist services and treatment

Around two thirds (166, 68.9%) of those found to have anti-HCV through the unlinked anonymous testing were estimated to have been tested and diagnosed up to the end 2017 (i.e. 11 months post the LFT sampling period) (Table 1). This was highest in those aged 37–50 (109, 83.2%), those with APRI score of one or greater (34, 82.9%) and those with ALT 50–69 (55, 73.3%). The majority of those ever diagnosed (141, 84.9%) had been diagnosed before the study period (i.e. pre 2016). This was highest among men (95, 88.0%), those aged 18–36 (20, 90.9%), those with ALT less than 30 (32, 97.0%), and within the smaller number of individuals with APRI data available, those with APRI score of less than 1 (48, 87.3%). PCR status at time of HCV diagnosis was available for 98.2% of diagnosed cases and 144 (86.7%) of diagnosed cases were PCR positive (indicating chronic infection).

Ever attendance at a specialist service among those diagnosed with chronic infection was high (133, 92.4%). Two-thirds (90, 67.7%) of those who attended specialist services had ever been treated. A total of 55 individuals were known to have achieved SVR, which represents a majority of those treated (61% of 90) but only a minority (38% of 144) of those who had received a chronic HCV diagnosis.

### Extent of undiagnosed and diagnosed infection requiring re-engagement

Of the 241 anti-HCV-positive individuals, 75 (31.1%) remained undiagnosed as at the end of 2017 (Table 2). Of 118 who had been diagnosed with chronic HCV and had not achieved an SVR pre-2016, 53 (44.9%) had not attended a specialist service during 2016–17. Thus the total number requiring assessment, and potentially treatment, for their HCV infection but were not engaged with specialist services during the study period was 128, representing more than half (53.1%) of those found to have anti-HCV. This was higher for those with ALT results of 50–69 among whom 45 (60%) were not engaged with services and for those aged 51–64 years where this accounts for a higher proportion (45, 66.2%).

**Table 2.** Extent of undiagnosed and diagnosed infection requiring assessment and potentially treatment for their HCV infection, but who were not engaged with specialist services, during the study period 2016–17

	Study population (2016–2017)		With anti-HCV (2016–2017)		Undiagnosed with anti-HCV (by end of 2017)		Ever diagnosed with chronic HCV (by end of 2017), excluding those who had achieved SVR pre 2016						Total number requiring assessment, and potentially treatment, for their HCV infection but were not engaged with specialist services during the study period (2016–17)		
							Total		Attended HCV specialist in 2016–17		Not attended HCV specialist in 2016–17				
	N	%	N1	% of N	N2	% of N1	N3 (N4 + N5)	% of N1	N4	% of N3	N5	% of N3	N6 (N2 + N5)	% of N	% of N1
Total	6007	100.0%	241	4.0%	75	31.1%	118	49.0%	65	55.1%	53	44.9%	128	2.1%	53.1%
Sex															
F	2832	47.1%	82	2.9%	24	29.3%	45	54.9%	26	57.8%	19	42.2%	43	1.5%	52.4%
M	3175	52.9%	159	5.0%	51	32.1%	73	45.9%	39	53.4%	34	46.6%	85	2.7%	53.5%
Age Group (in years)															
18–36	943	15.7%	34	3.6%	12	35.3%	17	50.0%	10	58.8%	7	41.2%	19	2.0%	55.9%
37–50	2116	35.2%	131	6.2%	22	16.8%	75	57.3%	40	53.3%	35	46.7%	57	2.7%	43.5%
51–64	2479	41.3%	68	2.7%	36	52.9%	24	35.3%	15	62.5%	9	37.5%	45	1.8%	66.2%
65+	468	7.8%	8	1.7%	5	62.5%	2	25.0%	0	0.0%	2	100.0%	7	1.5%	87.5%
Deprivation status for the locality of the GP practice(2)															
Quintile 1 (most deprived)	4668	77.7%	189	4.0%	62	32.8%	88	46.6%	49	55.7%	39	44.3%	101	2.2%	53.4%
Quintiles 2–5	1321	22.0%	42	3.2%	13	31.0%	21	50.0%	11	52.4%	10	47.6%	23	1.7%	54.8%
ALT Level															
<30	2310	38.5%	53	2.3%	20	37.7%	14	26.4%	10	71.4%	4	28.6%	24	1.0%	45.3%
30–49	700	11.7%	25	3.6%	10	40.0%	12	48.0%	6	50.0%	6	50.0%	16	2.3%	64.0%
50–69	1756	29.2%	75	4.3%	20	26.7%	42	56.0%	17	40.5%	25	59.5%	45	2.6%	60.0%
70+	1241	20.7%	88	7.1%	25	28.4%	50	56.8%	32	64.0%	18	36.0%	43	3.5%	48.9%
APRI Score															
<1	3012	50.1%	99	3.3%	28	28.3%	48	48.5%	29	60.4%	19	39.6%	47	1.6%	47.5%
1+	380	6.3%	41	10.8%	7	17.1%	29	70.7%	15	51.7%	14	48.3%	21	5.5%	51.2%

### HCV test uptake among patients with abnormal ALT during 2016–17

Of the 2997 individuals who had an ALT result of 50 or higher, 908 (30.3%) were tested during 2016–17 (Table 3). This was highest for those who had been diagnosed before 2016 and had not achieved an SVR (104, 73.1%), those with an APRI score of 1 or greater (170, 45.2%) and lowest for those with an ALT result of 70+ (248, 20.0%), those with an APRI score of less than 1 (294, 25.4%) and those aged 65+ (65, 25.7%).

**Table 3.** HCV test uptake among the study population with an abnormal liver function test result (ALT result greater than 50) in primary care, during 2016–2017

	Total patients with and ALT >50	Tested for HCV during study period (2016–17)	
		N	%
Total	2997	908	30.3%
Sex			
F	1059	353	33.3%
M	1938	555	28.6%
Age Group (Missing = 1)			
18–36	421	140	33.3%
37–50	1035	365	35.3%
51–64	1287	338	26.3%
65+	253	65	25.7%
SIMD (Missing = 16)			
1 (most deprived)	2135	659	30.9%
2 to 5	846	241	28.5%
ALT Level			
50–69	1756	396	22.6%
70+	1241	248	20.0%
APRI.Cat (missing = 1749)			
<1	1158	294	25.4%
1+	376	170	45.2%
HCV Status			
Diagnosed and SVR before 2016	15	10	66.7%
Diagnosed before 2016 and no SVR	104	76	73.1%
Tested before 2016 (not diagnosed)	837	234	28.0%
Not Tested for HCV before 2016	2014	588	29.2%

## Discussion

### Principal findings

This study examined the extent of HCV infection among individuals undergoing LFTs in primary care – particularly practices located in high deprivation areas – to gauge the potential of HCV testing in this population, beyond that recommended in current guidance (i.e. those with an unexplained abnormal LFT), to increase HCV diagnosis/re-diagnosis and engagement/re-engagement in treatment in the DAA era. Among 6007 study participants, the overall HCV antibody prevalence was 2.3% and 5.1% among those with normal and abnormal LFT results, respectively. The majority of the study population reside in areas of high deprivation, based on the location of the GP practice, and among this group HCV prevalence was appreciably high among those aged 37–50 with normal LFT results (4.0%) and with abnormal LFT results (9.5%) as well as those with abnormal results aged 18–36 (5.5%) and aged 51–64 (3.5%). Of the 241 found to be anti-HCV positive, 59% had been first diagnosed before the study period and 10% during the study period (i.e. around the time of the LFT test), with 31% in total (and similarly 28% among those with abnormal LFT) remaining undiagnosed.

While DAA treatments were licenced during the study period, these were initially prioritised in Scotland to those with advanced fibrosis/cirrhosis. They did not become available on an unrestricted basis in NHS Greater Glasgow & Clyde until 2017 and interpretation of treatment uptake and SVR rates should be interpreted in this context. While more than 90% of those diagnosed with chronic HCV had ever attended a specialist HCV treatment service by end of the study period, two-thirds had not cleared their virus through treatment (including a small proportion unsuccessfully treated before the availability of DAA therapies). Just over half (53%) of all HCV antibody positives required assessment, and potentially treatment, for their HCV infection but were not engaged with specialist service during the study period. The majority (58%) of HCV-infected individuals who were tested for LFTs during the study period had already been diagnosed with their infection prior to their LFT test (rising to 82% for those with abnormal LFTs). However, a minority (25%) of those previously undiagnosed were tested/diagnosed around the time of the LFT test (equivalent of 30% for those with abnormal LFT) and only 9% of those previously diagnosed (and who had not cleared their infection either spontaneously or via therapy) were re-tested/re-diagnosed at the same time or following the LFT test (equivalent of 7% for those with abnormal LFT).

### Validity of the observations

LFTs are among the most commonly requested tests in primary care and while the majority of results are normal, investigation and referral for patients with abnormal results is often insufficient [17, 18]. As shown in online appendix 1, our study cohort represents just over 10% of all the LFT requested in primary care at the same laboratories over a six-month period. Samples were selected for inclusion to ensure that half had an ALT result over 50 (compared with around 8% of all LFT results). Our cohort, therefore, is not representative of everyone who had LFTs based on the cohort selection as it differs from the denominator in terms of deprivation and ALT result and should be interpreted with this in mind. NHS Greater Glasgow and Clyde is the largest NHS area in Scotland in terms of population, contains some of the most deprived areas in the country and the highest prevalence of HCV [17–19]. Other areas will have variable HCV prevalence rates among patients with abnormal LFT results than described here. The use of data linkage using limited patient identifiers to ascertain HCV testing, diagnosis and treatment among the study cohort carries the possibility for error and there may be patients who have been matched incorrectly and others that have not been matched despite belonging to the same person [20]. This is an unavoidable limitation of a study of this nature. However, we have demonstrated an approach to developing criteria for HCV screening following LFT results based on age group and deprivation which can be adapted by other areas.

### Comparison with existing literature

The Scottish Intercollegiate Guidelines Network (SIGN) guidance for HCV recommends that ‘patients with an otherwise unexplained persistently elevated alanine aminotransferase’ should be offered an HCV test, which has been the longstanding recommendation since the guidance was published in 2006 [21]. In our study, we found that only 30% of those with ALT levels greater than 50 were tested for HCV during the study period; whilst we do not have information regarding their explained or unexplained status or whether the abnormal ALT results are persistent, the low proportion suggests concerted effort to increase HCV testing following an elevated LFT result is needed.

The Birmingham and Lambeth Liver Evaluation Testing Strategies (BALLET) cohort study followed up 1340 patients with abnormal LFT results in primary care who did not have a pre-existing/obvious liver disease for two years following the initial result [22]. That study found considerably lower HCV prevalence than we have described at 1.1%. However, this study considered the full spectrum of liver disease, including alcoholic liver disease and non-alcoholic fatty liver disease, rather than specifically HCV and focused on patients with ‘mildly’ abnormal results and excluded those who had a pre-existing or obvious liver disease. They were, therefore, less likely to identify those with HCV than in our study and would not identify any past HCV diagnoses who may require assessment as we have been able to demonstrate. That study recommended testing for viral hepatitis in those with obvious clinical indication of risk, those who originated in high prevalence countries and those who have ALT levels more than twice the upper limit of normal [23]. This accounts for the lower HCV prevalence observed as our cohort has been selected to include patients of higher risk.

Algorithms to generate probable diagnoses of liver disease following LFT testing have been developed in NHS Tayside, another area of Scotland with deprivation and HCV prevalence higher than the national average [24, 25]. These highlight the importance of correct follow up of patients with otherwise unexplained abnormal LFTs to diagnose liver disease, and in particular the benefits of reflex testing of samples.

Reflex testing, where an additional test is automatically undertaken on a sample when clinically relevant, has been identified as a method of improving HCV diagnosis by automatically testing all HCV antibody-positive samples for PCR to determine chronic status rather than requesting a second sample [26, 27]. With proper consent at the time of the LFT sampling, i.e. that the patient knows that their blood may be tested for HCV if they meet the criteria, this could streamline HCV case-finding efforts and should be considered. Reflex testing has been demonstrated as an effective method of increasing HCV case-finding in hospital settings in other countries but this is the first study to demonstrate the utility of reflex testing in primary care [28–30].

### Significance of research

Since the availability of affordable DAA therapy for HCV, there has been a fundamental shift in the approach most resource-rich countries take to tackle the epidemic with the aim of elimination of HCV as a public health concern. This has led to more focus on efforts to increase case-finding and re-engagement of those who are lost to follow-up. Initiatives, such as the introduction of Dried Blood Spot (DBS) testing in addiction services and opt-out testing in prisons, have been developed to address this in key populations with increased risk of HCV [13, 31]. However, the biggest challenge for case-finding is the large proportion of patients who acquired their infection through injecting drugs in the past or those who acquired their infection through non-injecting routes [32]. Not only are those who fall into these groups less likely engage with the services specifically aimed at people who inject drugs (PWID), they may also be less likely to be asked about or disclose past risk behaviours. The use of LFTs as a biological marker to prompt an HCV test as the first step on a diagnostic algorithm for liver disease overcomes this barrier.

One third of HCV antibody-positive individuals had not been diagnosed by the end of 2017, eleven months after the end of the study. In a system where reflex testing following abnormal results had been implemented, 60% of these patients would have been newly diagnosed following their LFT test. Where the implementation of reflex testing is not possible, for example where the biochemistry labs are not able to undertake virology testing, a recommendation that the requesting clinician offers the patient an HCV test should be included with abnormal LFT results. In this study, among areas of the highest deprivation, those with normal LFT results (ALT<50) and aged 37–50 years had an HCV prevalence of 4.0%. This exceeds the 2% prevalence required for universal screening for HCV to be cost-effectiveness as recommended by the World Health Organization (WHO) testing guidance [33]. A universal programme for all those residing in the most deprived area and born between 1965 and 1980 (as those between 37 and 50 years of age in this study are), regardless of their LFT results could therefore be considered.

A key finding of this study was that less than 10% of patients who had previously been diagnosed with chronic HCV and who had not cleared their infection either spontaneously or through treatment had been re-tested for HCV following their LFT test. While re-testing for HCV antibodies in previously diagnosed patients would not be recommended, a PCR test to confirm chronic status should be undertaken. This represents a missed opportunity to re-engage and treat such individuals, particularly for those who were diagnosed before the availability of DAA treatment or those with evidence of liver damage.

## Conclusion

To achieve the WHO goal to eliminate hepatitis C as a public health concern, further efforts to increase diagnosis, engagement and treatment are required in most resource-rich countries. Our data highlight the appreciable prevalence of HCV among those with abnormal LFT results, in line with Scottish and international testing guidance, with particularly high prevalence observed in areas of high deprivation (ranging from 2.0% in those aged 65+ with ALT greater than 50 to 12% in those aged 31–50 with ALT levels greater than 70). Indeed, HCV prevalence among those residing in areas of high deprivation with normal LFT results was sufficiently high (2.6%) to consider expanding the testing guidance to include this cohort along with all those with abnormal LFT results. While the majority (69%) of those infected had been diagnosed with HCV in the past, we found that most had not cleared their infection through therapy and thus the LFT test offers a key opportunity to re-diagnose and engage those individuals. Finally, we found that only a minority of those found to have abnormal LFTs go on to receive an HCV test, despite the clear guidance in this area. Liver function tests in primary care are a key opportunity to diagnose, re-diagnose and re-engage patients with HCV infection and highlight the importance of GPs in efforts to eliminate HCV as a public health concern.

## Data Availability

Limited, anonymised data used in this study are available from the corresponding author on reasonable request.

## Supplementary material

For supplementary material accompanying this paper visit http://doi.org/10.1017/S0950268822000978. click here to view supplementary material
